# Refractory gastroesophageal reflux disease

**DOI:** 10.1093/gastro/gou061

**Published:** 2014-09-30

**Authors:** Charumathi Raghu Subramanian, George Triadafilopoulos

**Affiliations:** ^1^Internal Medicine, Guthrie Clinic, Sayre, PA, USA and ^2^Division of Gastroenterology, Stanford University School of Medicine, Stanford, CA, USA

**Keywords:** gastroesophageal reflux disease, acid-related diseases, pH monitoring, endoscopy

## Abstract

Gastroesophageal reflux disease (GERD) is a condition that develops when the reflux of stomach contents into the esophagus causes troublesome symptoms, esophageal injury, and/or complications. Use of proton pump inhibitors (PPI) remains the standard therapy for GERD and is effective in most patients. Those whose symptoms are refractory to PPIs should be evaluated further and other treatment options should be considered, according to individual patient characteristics. Response to PPIs could be total (no symptoms), partial (residual breakthrough symptoms), or absent (no change in symptoms). Patients experiencing complete response do not usually need further management. Patients with partial response can be treated surgically or by using emerging endoscopic therapies. Patients who exhibit no response to PPI need further evaluation to rule out other causes.

## INTRODUCTION

Gastroesophageal reflux disease (GERD) is defined as a condition that develops when the reflux of stomach contents into the esophagus causes troublesome symptoms, esophageal injury, and/or complications. GERD is estimated to affect 10-20% of adults in Western countries, on a daily or weekly basis [1]. GERD results from disruption of the anti-reflux barrier, composed of the lower esophageal sphincter (LES) and the diaphragmatic crura. Relaxation of the crura and LES is a normal physiological process that takes place during swallowing. Relaxations not initiated by swallowing are termed transient lower esophageal relaxations (TLESRs); when they occur more frequently or last longer, they result in reflux of gastric fluid through the esophagogastric junction (EGJ), sometimes accompanied by gas (belch). TLESRs contribute to almost 90% of reflux episodes; more severe reflux-induced esophageal damage (esophagitis) results from persistently hypotensive LES [2, 3].

**Figure 1.**
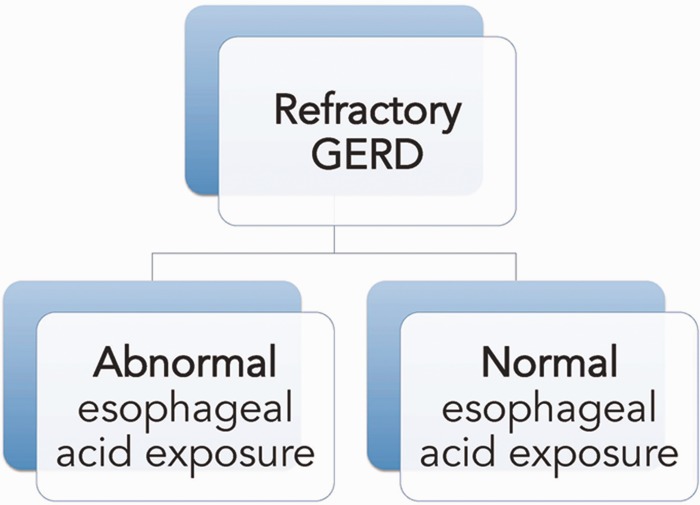
Refractory gastroesophageal reflux disease may be distinguished by the magnitude of esophageal acid exposure.

Heartburn and acid regurgitation, sometimes accompanied by chest pain and dysphagia, are the two cardinal symptoms of GERD but may reflect other, non-GERD conditions without abnormal (pathological) esophageal acid exposure (Figure 1). GERD could be erosive or non-erosive. Erosive esophagitis (EE) is defined as reflux-induced inflammation or ulceration of the esophagus. Patients who do not have erosive changes on endoscopy but have pathological amounts of acid reflux on 24-hour ambulatory pH monitoring are classified as suffering from non-erosive reflux disease (NERD). Other patients may have typical reflux symptoms without evidence of pathological acid reflux by pH monitoring and they are classified as suffering from functional heartburn [4]. In some patients, when acid reflux damages the esophageal squamous epithelium, the injury leads to a metaplastic process in which the squamous cells are replaced by columnar epithelium containing goblet cells (intestinal metaplasia). This is called Barrett’s esophagus (BE), and is a precursor of esophageal adenocarcinoma.

## NON-GASTROESOPHAGEAL REFLUX DISEASE CAUSES

**Figure 2.**
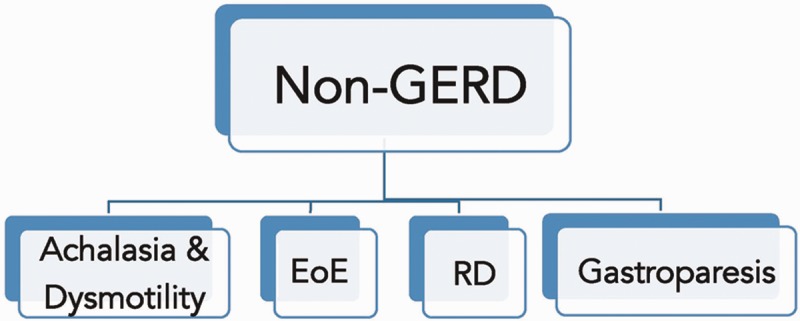
Non- gastroesophageal reflux disease causes of refractory gastroesophageal reflux symptoms.
- Achalasia & dysmotility: defined manometrically.- EoE (Eosinophilic Esophagitis): >15 eosinophils / HPF (high-power field).- RD (reflux-like dyspepsia): normal endoscopy, biopsies and pH monitoring.- Gastroparesis: normal endoscopy, abnormal gastric emptying scintigraphy.

Some patients experience symptoms of reflux as a reflection of other, non-GERD causes, such as gastroparesis, reflux-like dyspepsia, achalasia and other esophageal dysmotility, or eosinophilic esophagitis; overlap between GERD and these conditions may also occur [5] (Figure 2). Gastroparesis, or delayed gastric emptying, can present with reflux symptoms. These patients generally have normal endoscopic findings and an abnormal gastric emptying scintigraphy. In a study comparing clinical characteristics of responders and non-responders of reflux patients on proton pump inhibitor (PPI) therapy, symptoms suggestive of gastroparesis significantly reduced the odds of being a responder [6]. Functional dyspepsia is defined, according to the Rome III criteria, as the presence of one or more of the following: post-prandial fullness, early satiety, heartburn and no evidence of structural disease to explain the symptoms, for the previous 3 months, with symptom onset at least 6 months before. It is a diagnosis of exclusion and other causes should be ruled out by additional testing. Achalasia is an esophageal motility disorder that is characterized by incomplete LES relaxation, increased LES pressure and esophageal body aperistalsis, leading to poor clearance and esophageal dilation. Apart from dysphagia to solids and liquids, patients with achalasia may experience heartburn and regurgitation. Achalasia and other esophageal motility disorders (e.g. esophageal spasm and ‘jackhammer’ esophagus) should be differentiated from GERD with a barium swallow and high resolution manometry. Eosinophilic esophagitis is a chronic immune/antigen-mediated disease characterized clinically by symptoms related to esophageal dysfunction, such as heartburn or dysphagia, and histologically by eosinophil-predominant inflammation (>15 eosinophils per high-power field) [7]. Since GERD could be associated with esophageal mucosal eosinophilia, it should again be ruled out by esophageal pH monitoring study or by obtaining repeat biopsies after two months of treatment with a PPI.

## PROTON PUMP INHIBITOR FAILURE

The critical role of acid in triggering heartburn has been established in many clinical trials. Intra-esophageal instillation of solutions with increasing pH was tried in a study [8], in which all patients experienced pain at pH 1 and 1.5 solutions, 80% had pain with pH 2 solutions and 50% had pain with pH 2.5–6 solutions, thus establishing acid as a major cause of heartburn [8]. Acid-suppressive therapy with PPI has thus become the treatment choice for GERD. However, despite the high efficacy of PPIs, approximately 30% of patients fail to respond, either partially or completely. Failure of GERD patients to respond to PPI has now become the most common presentation of GERD in gastrointestinal practice [9]. Some authors consider refractory GERD as the failure to respond to the standard PPI regimen (once daily), while others believe that only patients who show incomplete or partial response to PPI twice daily should be considered as failures [10, 11]. The differential response of various GERD symptoms to PPI also makes it difficult to define PPI failure. In some patients, it has been shown that regurgitation is less responsive to acid suppression than heartburn, and that regurgitation is likely to be an important factor in determining response to PPI [12, 13]. PPI response also varies between different GERD types. In a Japanese study, rabeprazole 10 mg daily was administered to 180 patients with GERD for 4 weeks. Complete relief of symptoms was achieved in 36% of NERD patients, compared with 55% of erosive GERD patients [14]. A similar conclusion was also obtained in another western study, which compared NERD and EE for their response to PPIs [9].

## REASONS FOR FAILURE OF PROTON PUMP INHIBITORS

An important cause of PPI failure is non-compliance to treatment, because of either inadequate dosing or poor timing [15]. In a study of 100 patients taking PPI for GERD, compliance was determined using a questionnaire about dosing habits and timing. Only 46% dosed optimally. Among the 54% who were dosed sub-optimally, 39% dosed more than 60 minutes before meals, 30% after meals, 28% at bedtime, and 4% as needed [16]. Non-adherence to lifestyle modifications could also hinder response to treatment.

In some patients, the occurrence of weakly acidic (pH 4–7) or weakly alkaline (pH >7) refluxate may be associated with regurgitation and atypical GERD symptoms, but it is not clear whether such pH alterations are responsible for the symptoms. Esophageal exposure to bile salts at non-acidic pH may cause heartburn, but the mechanism remains unclear [17]. The volume of reflux could be a factor to consider, as it affects the intraluminal distribution and degree of esophageal distension. Greater reflux volume can trigger heartburn by mechanical distension, irrespective of its acidity. Esophageal balloon distension causes the sensation of heartburn in patients with GERD, and the likelihood of such heartburn increases in a linear fashion with increases in balloon volume [18]. A recent study compared GERD-related esophageal distension before and during PPI therapy. The majority of episodes during treatment were non-acidic; however, the acid suppression did not alter the magnitude of GERD-induced esophageal distension, suggesting that the remaining symptoms while on treatment could be due to the latter [19]. The volume of reflux also determined the proximal extent of reflux that is, in turn, associated with symptomatic episodes. Esophageal hypersensitivity is another proposed mechanism for symptom induction in patients with normal acid exposure on pH monitoring. Closely spaced reflux episodes were also more likely to be associated with severe symptoms than isolated reflux episodes [20]. NERD patients generally belong to a more hypersensitive group than those with EE, exhibiting a lower response to PPIs [21]. Other co-existing conditions, like obesity and *heliobacter pylori* infection are also less frequent in responders than non-responders [5]. Table 1 outlines various risk factors for refractory GERD.

**Table 1.** Risk factors for refractory gastroesophageal reflux disease

Increased body mass index
Sliding hiatal hernia
Increased frequency of gas, liquid or volume reflux
Ineffective PPI-induced control of gastric acid secretion
Pepsin and bile acid content of gastric juice
Hypotensive lower esophageal sphincter
Ineffective esophageal peristalsis
Low post-reflux swallow-induced peristaltic wave index
Esophageal hypersensitivity (central or peripheral)
Ultrastructural and functional changes in the esophageal epithelium

LES = lower esophageal sphincter; PPI = proton pump inhibitor.

## EVALUATION OF REFRACTORY REFLUX SYMPTOMS

**Figure 3.**
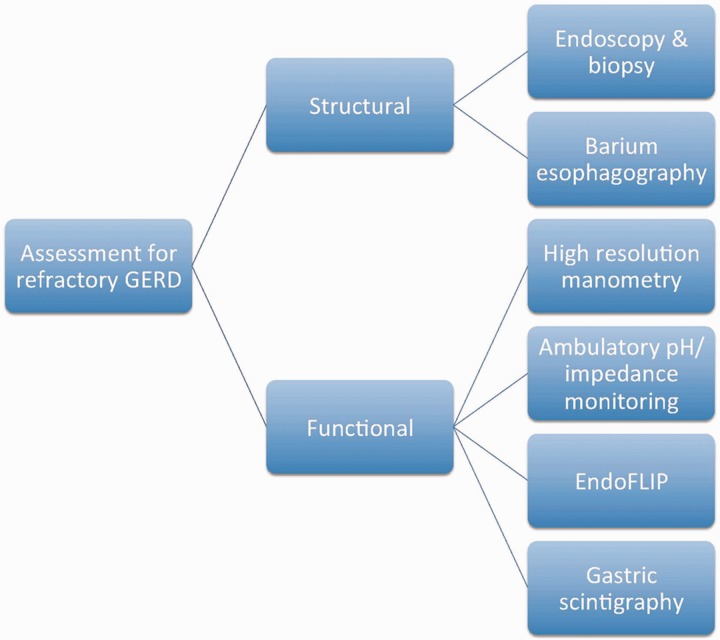
Structural and functional assessment of patients with refractory gastroesophageal reflux disease.

The first step in the evaluation of a patient who has failed to respond to PPI therapy is to assess drug compliance and the adequacy of lifestyle modifications. The next step is to switch to another PPI or increase the dose to twice daily. When these measures fail, further investigations are usually required (Figure 3). GERD could result from a structural or functional defect in the esophagus. The structural assessment can be done by endoscopy with biopsy, and barium esophagography. Functional assessment can be accomplished using high-resolution manometry (HRM), ambulatory impedance-pH monitoring, endoluminal functional lumen imaging probe (EndoFLIP), and gastric scintigraphy.

In patients with persistent symptoms despite treatment, the value of upper endoscopy is limited, since most patients have NERD or functional heartburn. However endoscopy could still be helpful in identifying the few cases of EE, BE or peptic ulcer, and also differentiate from other non-GERD causes, like eosinophilic esophagitis, cancer, etc. Additionally, esophageal histology could reveal the presence of dilated distal intercellular spaces, which have been put forward as a mechanism for symptoms of GERD [22]. A recent study confirmed the utility of magnification endoscopy with narrow-band imaging (NBI), a technique that enhances the microvascular and mucosal patterns not usually visible with normal white-light endoscopy. However, inter- and intra-observer agreement needs to be evaluated with further studies [23].

Ambulatory esophageal pH monitoring, either catheter-based (24 hours) or wireless (48 hours or more), can be performed while patients carry out their usual activities and eat normally. Such technologies allow pH testing to be performed both ‘off’ and ‘on’ PPI, ‘off’ therapy testing to determine if symptoms are truly due to reflux, and ‘on’ therapy testing to investigate whether there is persistent abnormal esophageal exposure despite PPI [24].

Esophageal impedance monitoring detects retrograde bolus movement and can determine the nature and proximal extent of reflux, regardless of acidity. Impedance is generally combined with a pH probe, which allows categorization of reflux into (i) acidic, (ii) weakly acidic or (iii) weakly alkaline. The addition of impedance monitoring to the routine pH monitoring allows correlation between symptoms and reflux episodes, and has been associated with a higher proportion of patients with symptom-association probability than with pH monitoring alone [25]. Whether the test is most beneficial when the patients are ‘off’ or ‘on’ therapy is debatable. One study, comparing the two approaches showed that, in patients ‘off therapy’ impedance-pH added only 4% to the results compared with pH testing alone whereas, in patients ‘on therapy’, there was a 17% increase in the diagnostic yield [26]. In contrast, another study concluded that a higher probability of positive symptom-association probability was among patients tested ‘off therapy’ and that impedance-pH monitoring should be performed after cessation of PPI [27].

HRM helps in the exclusion of motor disorders, like achalasia, and also assesses for ineffective esophageal peristalsis, which plays an important role in the induction of refractory reflux symptoms. It is a recently introduced technique that uses multiple, closely spaced sensors to measure the intraluminal pressure of the entire esophagus during swallowing. A new classification of esophageal motor disorders, the Chicago Classification, has been developed using several esophageal pressure topography metrics, constructed from HRM data. HRM-based studies improved both EGJ and TLESRs assessment and underlined their role as primary mechanisms in the development of reflux events [28]. Recent studies have indicated that HRM is reproducible and more sensitive than stationary manometry in detecting TLESRs associated with reflux [29]. Volume reflux is another concern with refractory GERD and could be measured by high-frequency intraluminal ultrasound probe (HFIUS). This experimental technique detects spontaneous GER-induced esophageal distension, and could estimate the cross-sectional area of the Esophageal lumen and the volume of refluxate [30].

Increased EGJ distensibility significantly affects the volume of reflux. The EndoFLIP system uses impedance planimetry to determine multiple adjacent cross-sectional areas within a cylindrical bag that is placed in the distal esophagus during volumetric distention. The procedure helps the identification of a group of patients with GERD whose symptoms—usually regurgitation and chest pain—are driven by large reflux volumes and would benefit from anatomical correction, by either surgical or endoscopic techniques. EndoFLIP may also identify a subgroup of patients with normal EGJ distensibility, which would not benefit from such interventions. Similarly, EndoFLIP measurements may be potentially useful in the course of surgical or endoluminal procedures, to calibrate the magnitude of the intervention.

## TREATMENT OF REFRACTORY GASTROESOPHAGEAL REFLUX DISEASE

**Figure 4.**
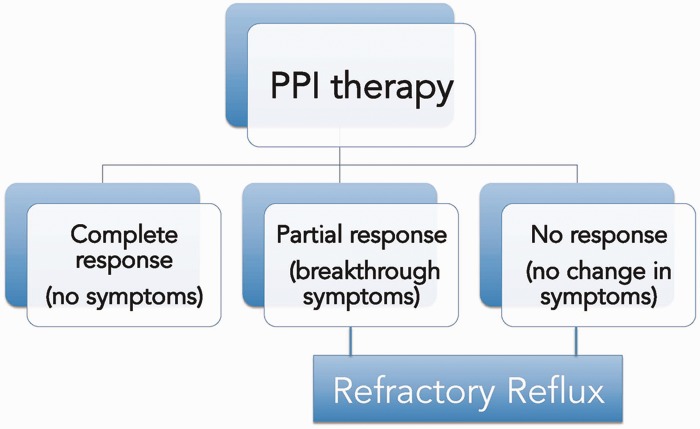
Potential outcomes of proton pump inhibitor (PPI) therapy in patients with gastroesophageal reflux symptoms.

Figure 4 illustrates a proposed treatment algorithm for patients with refractory GERD. After confirming the diagnosis of GERD, alterations in PPI therapy (type, dosage, timing) is the first step. Other medications (prokinetics and H2 receptor antagonists) could also be tried. Further management depends on the patient's response to PPIs. When there is complete response—that is, no residual symptoms while on PPIs—no further treatment is needed. When there is no response to PPIs, causes other than GERD should be considered. If there is partial response—that is, some improvement in symptoms—a variety of approaches could be entertained. At this point, two important factors need consideration: first, if the patient has significant sliding hiatal hernia (>3 cm), hernia repair, along with anti-reflux surgery, is currently the ideal option. Second, if the patient is morbidly obese, gastric bypass surgery may be the preferred approach. In patients without significant hiatal hernia (<2 cm) endoscopic therapies could be considered.

### Medical management

#### Proton pump inhibitors

When a patient has failed to respond to once-daily PPI, two types of modifications could be considered. The American Gastroenterological Association (AGA) guidelines for GERD recommend doubling the dose of the current PPI [31], and this is also confirmed by a Cochrane review for patients with esophagitis [32]. The doubled PPI dose can be divided, as one dose in the morning and one in the evening before meals, rather than a single higher dose. In a study examining nocturnal acid breakthrough, omeprazole, even at a moderately high dose, did not control acidity in most subjects for a 24-hour period and nocturnal re-acidification of the stomach typically in the early morning hours could be reduced by a divided dose of PPI, in the morning and evening before meals [33]. However the dose–response relationship for symptom control with PPIs is not clear [31]. Further, increasing PPIs beyond double the dose is not supported by clinical data. In a recent study, typical GERD symptoms, male gender and obesity were identified as good predictors of response to PPIs; in such patients, empirical PPI therapy could be successful. In the remaining patients, impedance pH monitoring should be considered instead of empirical PPI therapy. In PPI-resistant GERD patients, such monitoring can facilitate a more tailored therapeutic approach and ensure the success of further escalating PPI therapy [34].

Extended-release PPIs have being widely studied. Dexlansoprazole-MR is a dual, delayed-release formulation of dexlansoprazole, with two types of granules that release the drug at different pH levels; thus the drug achieves dual peaks in the serum, 1–2 hours after and 4–5 hours after administration. This offers greater dose flexibility and improved compliance. Although its use in PPI-refractory GERD has not been adequately researched, it may help as a step-down therapy from twice-daily PPI [35]. Succinic acid has been shown to induce gastric acid secretion. VECAM VECTA Pharmaceuticals, Ramat Gan, Israel (VECAM) is a combination of omeprazole and succinic acid that activates proton pumps in parietal cells. An open-labeled, randomized, crossover study enrolling 36 healthy subjects compared the effect on gastric acidity of once-daily bedtime dosing of VECAM 40 and VECAM 20 without food *vs.* omeprazole 20 mg administered before breakfast [36]. The median percentage time that the intragastric pH >4 demonstrated that VECAM 40 was superior to VECAM 20 (65.7% *vs.* 49.1%; P < 0.0001) and omeprazole 20 mg (65.7% *vs.* 47.6%, *P = *0.005) during 24 h.

A recent study evaluated the efficacy of pantoprazole magnesium in the treatment of GERD symptoms, especially at night. Pantoparazole magnesium has lower maximum plasma concentration than the conventional pantoprazole sodium and thus a longer half-life. Pantoprazole-Mg 40 mg once daily for 4 weeks improved a broad range of GERD-associated symptoms from baseline, including both day- and night-time GERD symptoms [37].

Switching to another PPI is another common and cost-effective approach for patients who failed to respond to PPI once daily. In a multicenter, double-blind, randomized, controlled study, switching to single dose esomeprazole was found to be as effective as twice-daily lansoprazole in controlling heartburn symptoms [38]. Esomeprazole, as an alternative for patients who did not respond favorably to other PPIs, has resulted in significant symptom improvement [39].

#### Other medications

When alterations in dosing and switching of PPI have failed, other drug therapies can be considered, depending on individual patient characteristics [40]. If impedance/pH monitoring is positive for acid reflux and PPI compliance has been confirmed, H2 receptor antagonists (H2RAs) can be considered at bedtime. H2RAs competitively block the interaction between H2 receptors and histamine on parietal cells and reduce pepsin and gastric acid volume [41]. The addition of H2RAs at night for patients on PPIs has been shown to improve symptoms; however, since patients may develop tolerance after a month of treatment [42], this approach should be preferably used on an as-needed basis.

If impedance/pH monitoring is positive for weakly acid reflux, TLESR reducers and pain modulators can be considered. A wide range of receptors is involved in triggering TLESRs, including GABA-B, cannabinoid, cholecystokinin (CCK), and 5-HT4 [43]. The effect of baclofen (a GABA-B agonist) on suppressing acid and non-acid reflux was measured by combined multichannel intraluminal impedance/pH, and was shown to significantly reduce the median number of post-prandial acid and non-acid related symptoms in heartburn patients [44]. However, since the drug crosses the blood brain barrier, central nervous system side-effects pose a challenge to its routine use. Gastrin and CCK2 receptors are identical and, considering the role of gastrin in the stimulation of gastric acid, a selective CCK2 receptor antagonist would be a potential therapeutic choice. Loxiglumide and spiroglumide are CCK2 receptor antagonists, but their use in humans has not yet been well validated [45]. Potassium-competitive acid blockers (PACB), such as soraprazan, linaprazan and revaprazan, share the same final mechanism as PPIs but in a reversible manner. They rapidly achieve high plasma concentrations and have linear, dose-dependent pharmacokinetics. They can be used as on-demand drugs [46].

Prokinetics, such as metoclopramide and domperidone, have been considered as a treatment option for GERD because they improve esophageal peristalsis, accelerate esophageal acid clearance, increase LES basal pressure and improve gastric emptying. Mosapride has both 5HT4 and 5HT3 receptor antagonist effects, and significantly reduces acid reflux. Itopride is a dopamine receptor antagonist that has also shown improvement in GERD symptoms. Unfortunately, prokinetics have been associated with several significant adverse effects and their use is GERD has been limited by concerns over safety [45]. Although combination therapy using PPI and prokinetics may partially improve patients’ quality of life, it has no significant effect on GERD patients' symptomatic or endoscopic responses [47].

Rebamipide has anti-inflammatory functions and may be effective as a mucosal protectant. In a randomized study, 60 PPI-refractory patients were assigned to either rebamipide or placebo for 4 weeks. At the end of drug administration, symptom scores did not differ between the two groups, although a significantly higher proportion of patients in the rebamipide group showed amelioration of abdominal pain and diarrhea [48].

Since the majority of PPI-refractory patients have either NERD-type or functional heartburn, pain modulators are a reasonable option. These include tricyclic antidepressants, trazodone, and selective serotonin re-uptake inhibitors [49]. Their efficacy has been demonstrated in double-blinded trials, especially in patients with hypersensitive esophagus [50, 51]. These visceral analgesics are used in non-mood-altering doses to counteract pain, and they currently provide a good alternative until improved, esophageal-specific therapies are available [52]. The side-effect profiles of these medications suggest only limited use, and indicate the need for development of safer drugs. Growth factors, such as epidermal growth factors and macrophage colony stimulating factors, have shown some effect in patients with GERD, but have not evolved to a reasonable option for therapy [53].

### Anti-reflux surgery

Anti-reflux surgery comprises a broad range of conventional and novel techniques designed to correct a mechanically defective sphincter and is recommended for patients who demonstrate partial (inadequate) response to PPI, or who cannot tolerate medical therapy. Clinical evidence suggests that anti-reflux surgery is more effective for patients with abnormal acid reflux, and the role of anti-reflux surgery in patients with normal esophageal acid exposure or acid sensitive esophagus needs to be explored further. A combination of symptoms, measurable indicators of the presence of abnormal reflux, and documentation of the motility of the esophagus and lower esophageal sphincter should be used in the assessment of patients undergoing anti-reflux surgery [54, 55]. A randomized, controlled trial compared the long-term effects of PPI against anti-reflux surgery for patients with chronic GERD and esophagitis. The proportion of patients in whom treatment remained effective after 7 years was higher in the surgical- than the medical group (66.7% *vs.* 46.7%, respectively; *P = *0.002) [56]. In patients with hiatal hernia ≥3 cm, hernia repair should be done along with anti-reflux surgery. This procedure is safe and effective and has low recurrence rate [57]. In morbidly obese patients, hernia repair can also be done concurrently and effectively with gastric bypass surgery [58]. Fundoplication is the conventional anti-reflux surgery. Other newer minimally invasive surgeries are LINX™ (Torax Medical, Shoreview MN, USA), and EndoStim™ (Endostim, Saint Louis, MO, USA).

#### Fundoplication

Fundoplication involves the construction of a peri-esophageal ring around the gastro-esophageal junction, buttressing the sphincter. This technique has evolved over the years and undergone many modifications. Laparoscopic Nissen fundoplication (LNF) is currently considered the standard procedure. Many randomized clinical trials have compared open and laparoscopic approaches, concluding that the laparoscopic approach results in fewer defective plications, incisional hernias or other complications, better respiratory function, reduced need for analgesics and shorter hospital stay [59, 60]. The Long-Term Usage of Esomeprazole vs Surgery for Treatment of Chronic GERD (LOTUS) trial compared LNF with an esomeprazole (20–40 mg daily) treatment group over 5 years. Although dysphagia and flatulence were more common in the surgical group, prevalence and severity of symptoms such as heartburn (16% in esomeprazole group *vs.* 8% in LNF group), and regurgitation (13% *vs.* 2%, respectively) were lower [61]. Patients with either NERD or EE may benefit equally from fundoplication.

As noted above, a major drawback with LNF is the high prevalence of post-operative dysphagia and gas bloat syndrome. To counteract this—and in contrast to the complete fundoplication, where the stomach is wrapped 360° around the esophagus—modifications were introduced, namely the anterior (LAF, Dor) and posterior (Toupet) partial fundoplications. With these techniques, the fundus is either laid over the top of the esophagus (anterior), or around the back (posterior). In a recent meta-analysis of five randomized, controlled trials, it was concluded that at 1 and 5 years, dysphagia and gas-related symptoms are lower after LAF than after LNF, and esophageal acid exposure and esophagitis are similar, with no differences in heartburn scores, patient satisfaction, dilations, and re-operation rate. These results lend Level 1a support for the use of LAF for the surgical treatment of GERD [62].

Toupet (LTF) or posterior fundoplication is a partial 270° dorsal wrap creating an adaptable reflux wrap, which allows normal physiological functions like burping and vomiting when necessary, and thus minimizes the complications of bloating and retching that are common with LNF. In a prospective, randomized study of 200 patients comparing LNF with LTF, over a 2-year follow up period, dysphagia was more common in patients undergoing LNF than for LTF (19 *vs.* 8; *P* < 0.05), while reflux control with LTF was as good as in LNF [63]. Another study followed up patients over a period of 7 years, comparing the results of Toupet and LNF. Seven of the patients (28%) who underwent Toupet procedures had severe GERD, a percentage comparable to the Nissen group (31.6%). The duration of operation, operative blood loss, morbidity, length of hospitalization, need for re-operation, and efficacy in terms of relieving symptoms (average follow-up* = *27.5 months) were comparable. It was concluded that Toupet is a safe and effective form of treatment for symptoms of GERD, including patients with severe form of disease [64]. Anterior and posterior fundoplications were also compared, and LTF was found to provide better control of reflux related symptoms, lower number of re-operations, and less need for antisecretory therapy [65, 66]. Currently LTF is considered the surgery of choice for normal-weight GERD patients qualifying for surgery [67].

Complications of fundoplication include dysphagia: early, which usually resolves within 3 months, and late (5%), which may require dilation or, rarely, revisional surgery. Early satiety, weight loss, discomfort with large meals, hiccup and difficulty burping or vomiting can also occur. Heartburn and regurgitation can recur in 10% of patients after 5–10 years of surgery. Revisional surgery may be needed in 2–5% of patients and is usually effective [68]. Data from studies regarding use of PPI after anti-reflux surgery have been self-contradictory. A recent study estimated that as many as 50% patients who underwent anti-reflux surgery became PPI users 10–15 years post-surgery [69].

#### Gastric bypass

Obesity plays a major role in the development of GERD, since an increased body mass index (BMI) may cause incompetence of the gastro-esophageal junction. A correlation between body weight and BMI has been demonstrated in a study of 30 morbidly obese people who underwent esophageal function testing. Patients with pathological acid exposure weighed more than those with normal acid exposure, and patients with abnormal reflux scores weighed more and had higher BMIs than those with normal scores [70]. Thus, treating obesity is an important step in the treatment of GERD. For patients with moderate obesity (BMI 35–40), LNF shows good symptom control and moderate weight loss. For patients with BMI >40, gastric bypass surgery is preferred [68]. This laparoscopic procedure involves the creation of a small gastric pouch and connecting it directly to the intestine, bypassing a major portion of the mid/distal stomach and duodenum.

The efficacy of various bariatric surgeries in the amelioration of GERD was studied in the Bariatric Outcomes Longitudinal Database. GERD score was graded on a 6-point scale pre- and post-operatively. GERD score improvement in gastric bypass patients was 56%, compared with adjustable gastric banding (46%) and sleeve gastrectomy (LSG) (41%) [71]. Favorable results were also obtained in another study that compared gastric bypass with sleeve gastrectomy, where 558 patients underwent gastric bypass or LSG. With the exception of GERD, similar comorbidity remission rates were observed between LSG and gastric bypass for all other parameters [72]. In a comparative study, laparoscopic gastric bypass was also found to be as safe as fundoplication for morbidly obese patients. The overall in-hospital complications were significantly lower in the bypass group, while the mean length of hospitalization, mortality and treatment costs were comparable [73]. Thus bariatric surgery, particularly gastric bypass, and the resultant sustained weight loss demonstrate a significant reduction in GERD symptoms, and should be considered in appropriate patients.

## THE GASTROESOPHAGEAL REFLUX DISEASE TREATMENT GAP

**Figure 5.**
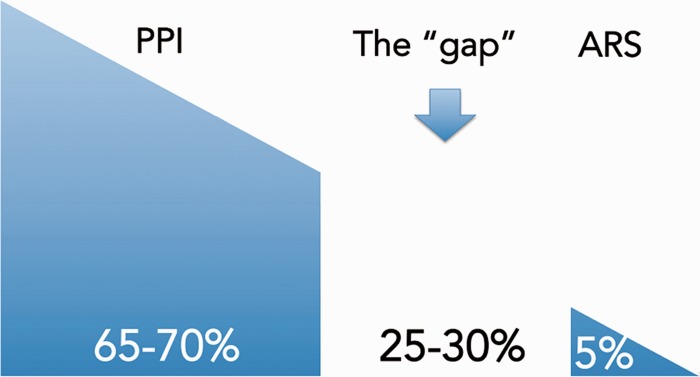
The treatment gap in patients with refractory gastroesophageal reflux symptoms. 'Gap’ is the percentage of patients refractory to proton pump inhibitor (PPI) not interested in undergoing anti-reflux surgery (ARS).

Studies show that, although approximately 40% of patients with GERD fail to respond symptomatically to aggressive acid suppressive therapy, less than 5% of them undergo fundoplication [74], leaving a substantial number of people receiving inadequate treatment for their GER symptoms (Figure 5). Such reluctance to proceed with surgery is partly due to the fear of possible side-effects from fundoplication, the reported high rates of surgical failures, and the subsequent need for medical therapy or repeat surgery. Patients who have persistent GERD symptoms despite medical therapy, and who are not willing to undergo fundoplication, fall into what is called the GERD ‘treatment gap’ (Figure 3). Newer techniques, surgical and endoscopic, have been introduced to address this gap. The advantage of these procedures is that they do not dramatically alter the anatomy of the gastroesophageal junction, esophagus or stomach, and thus have a better side-effect profile.

## SURGICAL APPROACHES TO THE GASTROESOPHAGEAL REFLUX DISEASE TREATMENT GAP

### LINX™

The LINX™ reflux management system mechanically augments the LES function, using a small expandable ring of linked magnetic beads. The device is laparoscopically implanted around the distal esophagus at the level of the EGJ. The magnetic attraction between each bead augments the pressure on the LES. At higher pressures, the magnetic forces are overcome, allowing functions such as swallowing, belching or vomiting [74]. The efficacy and safety of this device has been recently evaluated prospectively in 23 patients with GERD, and a significant decrease in all major GERD symptoms was found. At 4 weeks, a reduction of >50% of PPI dose was achieved in over 80% of patients. There were no serious adverse events. GERD-related quality of life improved significantly [75]. Long-term clinical benefits were demonstrated in a multicenter, prospective study where 44 patients with GERD underwent the procedure. For esophageal acid exposure, the mean total percentage of time that pH is less than 4.0 was reduced from 11.9% at baseline to 3.8% at 3 years (*P*<0.001), with 80% of patients (18/20) achieving pH normalization. In patients with more than 4 years of follow up, 100% had improved quality of life measures for GERD and 80% had achieved complete cessation of the use of PPIs, with minimal side-effects and no safety issues [76]. In another prospective case series, 100 patients were evaluated, who underwent the procedure over a period of six years. Median total acid exposure time was reduced from 8.0% before implant to 3.2% post-implant (*P* < 0.001). The median GERD health-related quality of life score at baseline was 16 on PPIs and 24 off PPIs and improved to a score of 2 (*P* < 0.001) and freedom from daily dependence on PPIs was achieved in 85% of patients [77]. Thus, magnetic sphincter augmentation is an effective and safe procedure for patients who fall in the treatment gap. The procedure does not significantly alter gastric anatomy and can be reversed if necessary. Randomized, controlled studies will be required, to clarify its efficacy as compared with LNF.

### EndoStim™

The EndoStim™ LES stimulation system (EndoStim, St. Louis, MO, USA) is an implantable electrical stimulator that delivers electrical energy to the LES. It comprises three components: a bipolar stimulation lead, an implantable pulse generator (IPG), and an external programmer. The stimulation leads are laparoscopically implanted in the LES and permanently secured, along with the IPG, in a subcutaneous pocket in the left upper quadrant of the abdomen. The external programmer allows for wireless interrogation and programming of the IPG [78, 79]. Electrical stimulation is believed to increase the resting pressure and control reflux.

### Lower esophageal sphincter electrical stimulation therapy

In an open-label, prospective trial, the safety and efficacy of long-term LES electrical stimulation therapy (LES-EST) was investigated. Patients with GERD were selected, who were at least partially responsive to PPI therapy, with hiatal hernia ≤3 cm, and with esophagitis Twenty-four patients underwent implantation. Median GERD-HRQoL score at 6 months was 2.0 (Interquartile range (IQR) = 0–5.5) and was significantly better than both baseline on-PPI [9.0 (range = 6.0–10.0); *P* < 0.001] and off-PPI [23 (GERD-HRQoL range = 21–25); *P* < 0.001] GERD-HRQoL. At their 6-month follow-up, 91% of the patients (21/23) were off PPI and had significantly better median GERD-HRQoL on LES stimulation, compared with their on-PPI GERD-HRQoL at baseline (9.0 *vs.* 2.0; *P* < 0.001). No serious adverse events were reported [79]. In a *post-hoc* analysis of the open-label trial, significant improvement in the outcomes of GERD-HRQoL and distal esophageal pH were noted in the LES-EST group. At baseline, 33% of PPI-treated patients reported nocturnal heartburn symptom “bothersome”, compared with 0% (*P = *0.04) at 3 months and 7% (*P = *0.17) at 6 months. In a more recent study, five patients successfully underwent implantation and all of them showed significant increase in LES pressure on all sessions of EST, without any adverse event [80]. In another *post-hoc* analysis, the effect of LES-EST on proximal esophageal acid exposure was studied, measured 23 cm above the upper border of the LES. Total median proximal esophageal acid exposure at baseline was 0.4% and, at 12 months, it was 0%. Distal esophageal pH improved from 10.2% to 3.6%. There were no serious adverse events. It was concluded that LES-EST might be useful in treating proximal GERD [81]. Hence, electrical stimulation of the LES is an effective alternative for patients who only partially respond to PPIs; its lack of any effect of esophageal motility or LES relaxation is an added advantage, especially in patients with poor esophageal motility [82].

## ENDOSCOPIC THERAPIES FOR THE GASTROESOPHAGEAL REFLUX DISEASE TREATMENT GAP

### Stretta®

Stretta® (Mederi Therapeutics, Greenwich, CT, USA) uses radiofrequency energy to remodel the EGJ and LES. The technology consists of a four-channel RF generator and a specialized balloon/catheter system that is used to treat the EGJ and cardia in a series of steps. Stretta has been shown to be effective and safe in 32 separate clinical studies and a meta-analysis [83]. A high rate of symptom control and a dramatic decrease in—or elimination of—GERD medication use, have been consistently achieved. As the endoscopic procedure with the most available data and track record, Stretta, if necessary, appears to be safe, effective, durable, and repeatable. Further, it does not preclude any alternative treatment (repeat Stretta, PPI addition or fundoplication) and is the least expensive alternative to medical therapy. Several putative mechanisms could explain Stretta’s clinical effectiveness and these include increased gastric yield pressure, increased thickness of the LES muscle, decreased distensibility of the EGJ without fibrosis, decreased EGJ compliance and decreased frequency of TLESRs.

A recent meta-analysis of 18 studies and 1488 patients concluded that (i) Stretta is very effective in GERD symptom relief, (ii) it is safe and well-tolerated and (iii) it significantly reduces acid exposure to the esophagus, but does not consistently normalize pH [84]. On this last point, it is important to note that even PPIs do not normalize pH in up to 50% of symptomatically controlled GERD patients [85]; hence, pH normalization is not necessarily an essential clinical endpoint to be applied to Stretta. In a single-center, 10-year, long-term study, normalization of GERD-related quality of life was achieved in 72% of patients; a 50% or greater reduction in PPI use occurred in 64% of patients (41% eliminating PPIs entirely) and a 60% or greater increase in satisfaction occurred in 54% of patients. Pre-existing Barrett's metaplasia regressed in 85% of biopsied patients [86].

A randomized, sham-controlled trial assigned 64 GERD patients to Stretta or to a sham procedure. At 6 months, active treatment significantly improved patients’ heartburn symptoms and quality of life. More active *vs.* sham patients were without daily heartburn symptoms (61% *vs.* 33%; *P = *0.05), and more had a >50% improvement in their GERD-HRQoL scores (61% *vs.* 30%; *P = *0.03) [87]. Another randomized prospective trial included 36 patients who were randomized into three groups: single-session Stretta, sham procedure, and single Stretta followed by repeat Stretta if GERD-HRQoL was not improved by 75% as compared to baseline GERD-HRQoL after 4 months. At 12 months, the mean HRQoL scores of those ‘off’ medications, the LES basal pressure, the 24 hour pH scores, and the PPI daily dose were significantly improved from baseline in both Stretta groups (*P* < 0.01). Seven patients in the double-Stretta treatment group had normalized their HRQoL at 12 months, compared with two patients in the single- treatment group (*P = *0.035) [88]. Like the other newer techniques, Stretta has not been found useful in patients with hiatal hernias >3 cm, those with no previous response to PPIs, or those with negative pH or impedance studies.

### Transoral incisionless fundoplication

Transoral incisionless fundoplication (TIF) is a newer technique devised to perform fundoplication endoscopically. The device retracts the gastric cardia and valve-like effect [89]. TIF was found to reduce the number of post-prandial TLESRs, the number of TLESRs associated with reflux, and EGJ distensibility, leading to a reduction of the number and proximal extent of reflux episodes and improvement of acid exposure. The anti-reflux effect of TIF proved to be selective for liquid-containing reflux only, thereby preserving the ability of venting gastric air [90].

Several studies have asserted the efficacy and safety of TIF. The TIF EsophyX vs Medical PPI Open Label Trial (TEMPO) trial was a multicenter, prospective, randomized, controlled study, in which 63 patients were randomized into the TIF or PPI groups. At 6-month follow-up, 62% of TIF patients reported elimination of regurgitation and atypical GERD symptoms, as against 5% in the PPI group; 90% of TIF patients were off PPI [91]. The long-term effects of TIF have also been evaluated prospectively in a multicenter study of 54 patients who underwent TIF and were followed up for 3 years. The median GERD-HRQoL score off PPI improved significantly and discontinuation of daily PPI use was sustained in 61% of patients. Although complete normalization of pH occurred in a minority of patients, successful cases showed long-term durability [92]. TIF has emerged as a safe, effective and durable alternative to GERD in patients who do not respond completely to PPI—without the adverse event profile associated with surgical fundoplication.

### Medigus

The SRS™ endoscopic stapling system (Medigus Ltd, Omar, Israel) is a recently introduced technique capable of creating an endoscopic partial fundoplication. The device consists of a flexible endoscope, a video camera, an ultrasonic rangefinder, and a surgical stapler. The SRS endoscope is inserted and advanced into the stomach and retroflexed, pulling it back to the correct stapling level above the EGJ. Tissue is then clamped and stapled under an ultrasonographic gap finder. The procedure is repeated a few times to form a flap, representing a 180^o^ fundoplication. The procedure has shown promise in a pre-clinical trial, where 12 study animals underwent the procedure, and all of them had a satisfactory partial fundoplication, with no procedure-related complications [93]. One of the first human trials of SRS was conducted recently to compare with Laparoscopic antireflux surgery (LARS). Of 27 patients, 11 underwent SRS and 16 underwent LARS. Over a 6-month follow-up period, a decrease in GERD-HRQoL scores was achieved in 64% and 87% of patients who had SRS and LARS, respectively. Larger randomized studies with longer periods of follow-up are required before its clinical use should be considered [94].

## CONCLUSIONS

**Figure 6.**
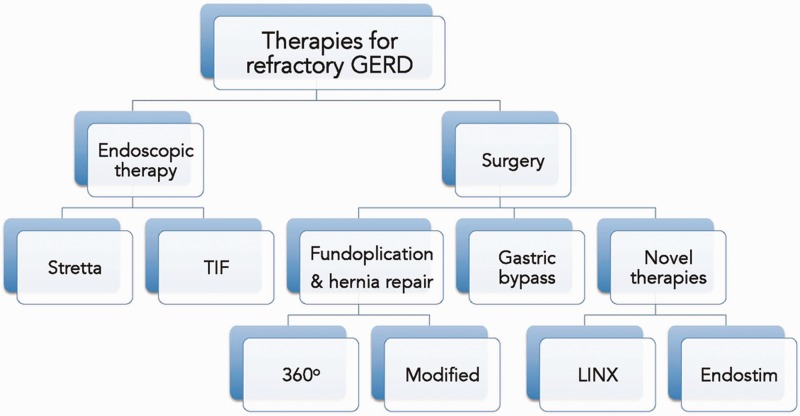
Available endoscopic and surgical therapies for refractory gastroesophageal reflux disease. TIF = transoral incisionless fundoplication.

PPIs remain the standard therapy for GERD and are effective in most patients. Those whose symptoms are refractory to PPIs should be further evaluated and other treatment options should be considered, according to individual patient characteristics (Figure 6). Response to PPIs could be complete (no symptoms), partial (residual breakthrough symptoms), or absent (no change in symptoms). Patients enjoying complete response usually do not need further management. Patients with partial response can be treated surgically or by using any of the emerging endoscopic therapies. The key features of the different treatment options for refractory GERD are outlined in Table 2. Patients who exhibit no response to PPI need further evaluation to rule out other causes.

**Table 2.** Key features of different treatment options for refractory gastroesophageal reflux disease

Therapy	Features
Stretta®	Outpatient; endoscopic; easy to perform; good long-term efficacy and safety; minimal side-effects
TIF	Outpatient; endoscopic; difficult to perform; good efficacy and safety; minimal side-effects
Fundoplication	Very effective; laparoscopic; very good long-term efficacy and safety; some long-term side-effects
Gastric bypass	Very effective; laparoscopic; limited applicability to morbidly obese; long-term nutritional side-effects
LINX®	Effective; laparoscopic; easy to perform; very good efficacy and safety; minimal side-effects; foreign body concerns
EndoStim®	Effective; laparoscopic; easy to perform; very good efficacy and safety; battery life issues; foreign body concerns

TIF = transoral incisionless fundoplication.

**Conflict of interest:** George Triadafilopoulos serves as an advisor to Mederi Therapeutics and Endostim.
